# 单中心阿扎胞苷联合维奈克拉和联合HAG方案诱导治疗老年急性髓系白血病的疗效比较

**DOI:** 10.3760/cma.j.issn.0253-2727.2023.09.011

**Published:** 2023-09

**Authors:** 新笑 卢, 琳玉 原, 凯奇 刘, 秋秋 张, 雪 王, 潇思 姜, 俊士 张, 邢力 赵

**Affiliations:** 1 天津市人民医院肿瘤血液科，天津 300121 Department of Hematology, Oncology Center, Tianjin Union Medical Center of Nankai University, Tianjin 300121, China; 2 南开大学医学院，天津 300071 School of Medicine, Nankai University, Tianjin 300071, China; 3 中国医学科学院血液病医院（中国医学科学院血液学研究所），天津 300020 Institute of Hematology & Blood Disease, Chinese Academy of Medical Sciences & Peking Union Medical College, Tianjin 300020, China

急性髓系白血病（AML）是异质性的血液系统恶性肿瘤，除疾病本身因素外，年龄是影响预后的重要因素[1]–[2]，研究显示，60岁以上AML患者4年总生存（OS）率显著低于60岁以下患者（16％对37％）[3]。本研究我们对本中心采用阿扎胞苷（AZA）+维奈克拉（VEN）方案与AZA+HAG方案诱导治疗老年AML患者的疗效、安全性及治疗费用进行比较，从而为我国老年初诊AML患者诱导治疗的选择提供经验。

## 病例与方法

1. 病例资料：回顾性分析天津市人民医院2020年6月至2022年10月收治的初治老年（≥60岁）AML患者临床资料，所有患者均参照WHO诊断标准[4]–[5]明确诊断为AML。所有患者根据情况均采用AZA+VEN或AZA+HAG诱导治疗。

2. 治疗方案：①AZA+VEN组：诱导化疗：AZA 75 mg·m^−2^·d^−1^，第1～7天；VEN 100 mg第1天、200 mg第2天、400 mg第3～28天。诱导缓解后，患者巩固及维持化疗同诱导化疗，直至疾病进展。②AZA+HAG组：诱导化疗：AZA 75 mg·m^−2^·d^−1^，第1～7天；高三尖杉酯碱（HHT）2 mg/d，第1～7天；阿糖胞苷（Ara-C）100 mg/d，第1～7天；G-CSF 300 µg/d皮下注射，直至WBC>10 × 10^9^/L时停用。巩固化疗：Ara-C 0.5 g，每12 h 1次，第1～3天，3个周期。维持化疗：AZA 75 mg·m^−2^·d^−1^，第1～7天，28 d为1个周期，直至疾病进展。

3. 疗效评估：疗效判定参照血液病诊断及疗效标准（第四版）[6]。复合缓解率为完全缓解（CR）率和伴血细胞未完全恢复的CR（CRi）率之和。不良反应（AE）标准参考CTCAE 5.0。粒细胞缺乏恢复时间、血小板恢复时间定义为脱离血制品输注，中性粒细胞>0.5×10^9^/L，PLT>20×10^9^/L。

4. 随访：以查阅住院、门诊病历及电话、微信等方式进行随访，随访截至2022年12月1日。

5. 统计学处理：应用SPSS 23.0软件进行统计学分析。计数资料组间比较采用Fisher确切概率法，计量资料组间比较采用独立样本*t*检验；OS及无复发生存（RFS）率根据Kaplan-Meier法计算，并描绘生存曲线；*P*<0.05为差异有统计学意义。

## 结果

1. 基本临床资料：共36例初治老年AML患者纳入研究，中位年龄为63（60～75）岁，中位WBC 15.2（0.7～373.0）×10^9^/L，中位PLT 49.5（5～240）×10^9^/L，参考2017年欧洲白血病网（ELN）预后分层，高危组14例（38.9％），中危组12例（33.3％），低危组7例（19.4％）；采用AZA+VEN诱导治疗22例，AZA+HAG诱导治疗14例。AZA+VEN组中位年龄及高危患者比例均高于AZA+HAG组，两组其他基线特征差异无统计学意义（表1）。

**表1:** AZA+VEN组和AZA+HAG组患者基线特征比较

特征	AZA+VEN组（22例）	AZA+HAG组（14例）	*P*值
年龄［岁，*M*（范围）］	65（60~78）	62.5（60~68）	0.015
性别［例（%）］			0.322
男	12（54.5）	5（35.7）	
女	10（45.5）	9（64.3）	
WBC［×10^9^/L，*M*（范围）］	13.4（1.2~373.0）	16.0（0.7~311.0）	0.618
PLT［×10^9^/L，*M*（范围）］	39.5（5~166）	67.5（6~240）	0.250
危险度分组［例（%）］			0.009
低危	4（18.2）	3（21.4）	
中危	8（36.4）	4（28.5）	
高危	9（40.9）	5（35.7）	
无法分组	1（4.5）	2（14.2）	
ECOG评分［例（%）］			0.712
0~1分	17（77.3）	10（71.5）	
≥2分	5（22.7）	4（28.5）	
基因突变［例（%）］			
FLT3-ITD	7（31.8）	4（28.6）	1.000
NPM1	6（27.3）	6（42.8）	0.471
DNMT3A	6（27.3）	5（35.7）	0.716
IDH1/IDH2	5（22.7）	3（21.4）	1.000

**注** AZA：阿扎胞苷；VEN：维奈克拉；HAG：高三尖杉酯碱+阿糖胞苷+G-CSF；ECOG：美国东部肿瘤协作组

2. 诱导治疗缓解率：AZA+VEN组总体复合缓解率为86.4％（19/22），1个疗程复合缓解率为81.8％（18/22），1个疗程微小残留病（MRD）阴性率77.3％（17/22）；AZA+HAG组总体复合缓解率为85.7％（12/14），1个疗程复合缓解率为71.4％（10/14），1个疗程MRD阴性率64.2％（9/14）。两组比较，总体复合缓解率、1个疗程复合缓解率及1个疗程MRD阴性率差异均无统计学意义（*P*值分别为1.000、0.683、0.462）。

3. 生存情况：截至2022年12月，所有患者中位随访时间为13（2～26）个月。AZA+VEN组中位随访时间为13（2～25）个月，随访期内中位OS时间未达到，中位RFS时间为17个月，1年OS率为75.8％，1年RFS率为69.0％；AZA+HAG组中位随访时间为14（6～26）个月，随访期内中位OS时间未达到，中位RFS时间为8个月，1年OS率为53.6％，1年RFS率为52.0％。两组OS及RFS差异均无统计学意义（图1）。

**图1:**
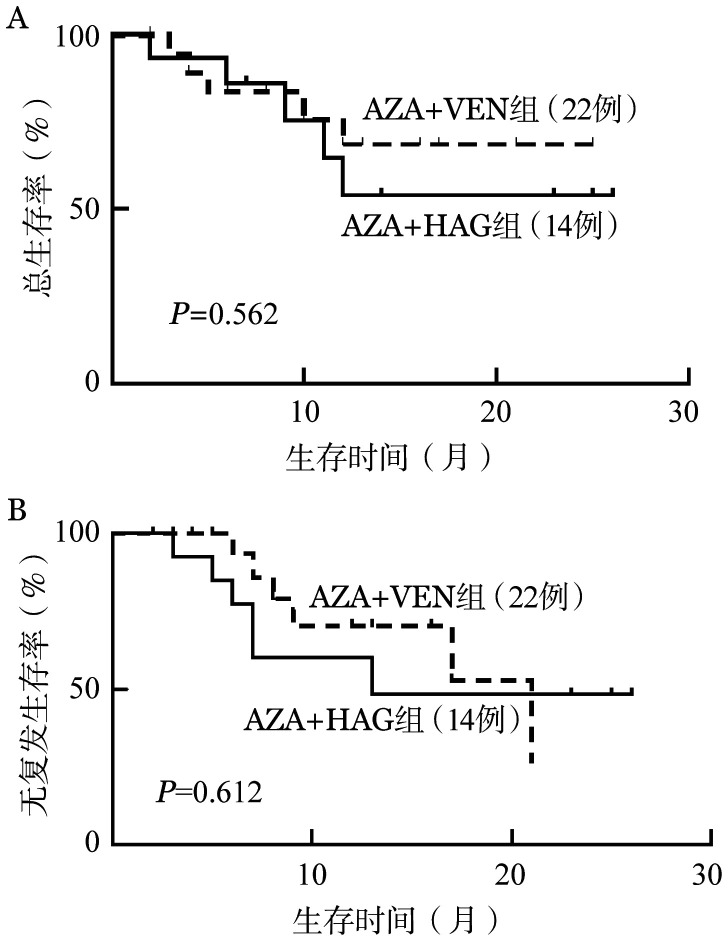
AZA+VEN及AZA+HAG治疗老年急性髓系白血病总生存（A）和无复发生存（B）的比较 **注** AZA：阿扎胞苷；VEN：维奈克拉；HAG：高三尖杉酯碱+阿糖胞苷+G-CSF

4. 安全性分析：

（1）血液学AE：两种治疗方案最常见的AE均为血液学AE。部分患者在治疗开始前已处于不同程度的血细胞减少，应归因于血液学疾病本身所致。两组患者的严重血小板减少比例、粒细胞缺乏比例、粒细胞缺乏恢复时间及血小板恢复时间见表2，AZA+HAG组严重血小板减少比例高于AZA+VEN组（*P*＝0.020）。

（2）非血液学AE：两组患者最常见的非血液学AE主要为骨髓抑制期发热、胃肠道不良反应、低钾血症及皮疹，两组非血液学AE发生率差异均无统计学意义。AZA+VEN组1例患者在治疗过程中出现急性冠脉综合征，予停用VEN，经吸氧、抗血小板聚集、扩冠等治疗后患者症状好转。两组患者治疗过程中均未观察到肿瘤溶解综合征的发生（表2）。

**表2:** AZA+VEN组及AZA+HAG组不良反应的比较

不良反应	AZA+VEN组（22例）	AZA+HAG组（14例）	*P*值
血液学不良反应［例（%）］			
粒细胞缺乏	10（45.5）	9（64.3）	0.050
严重血小板减少	9（40.9）	8（57.1）	0.020
粒缺恢复时间［d，*M*（范围）］	6（0~21）	12（0~26）	0.061
血小板恢复时间［d，*M*（范围）］	7（0~62）	11（0~87）	0.139
非血液学不良反应［例（%）］			
骨髓抑制期发热	12（54.5）	8（57.1）	1.000
恶心和呕吐	12（54.5）	8（57.1）	0.733
便秘	10（45.5）	6（42.9）	0.878
腹泻	11（50.0）	6（42.9）	0.742
肠梗阻	1（4.5）	0（0）	1.000
消化道出血	0（0）	1（7.1）	0.389
皮疹	2（9.1）	4（28.6）	0.181
心脏毒性	1（4.5）	0（0）	1.000
低钾血症	4（18.2）	4（28.6）	0.683

**注** 粒缺：粒细胞缺乏

（3）治疗相关死亡（定义：诱导治疗30 d内死亡）：AZA+VEN组2例死亡，死亡率9.1％，死因分别为败血症和多器官功能障碍；AZA+HAG组1例死亡，死亡率为7.1％，死因为呼吸衰竭。两组患者治疗相关死亡率差异无统计学意义（*P*＝1.000）。

5. 住院费用对比：AZA+VEN组诱导化疗中位住院费用为132 468.85（71 068.18～21 695.74）元，AZA+HAG组诱导化疗中位住院费用为98 724.63（46 475.7～15 3721.26）元，与AZA+HAG组比较，AZA+VEN组住院费用明显升高（*P*＝0.027）。

## 讨论

AML在老年人中发病率高，治疗效果差，不适合强化疗的老年AML患者较难从传统化疗中受益[7]。去甲基化药物是老年AML常用的治疗药物之一，该药物在提高患者缓解率的同时，能够一定程度改善患者的远期预后，但缓解率的提高相对有限[8]；研究发现，如能获得缓解，老年AML的生存时间可获得显著延长。因此，目前老年AML治疗方案改进的热点之一是在去甲基化药物的基础上，联合应用其他化疗药物或靶向药物，以期在具有良好耐受性的同时，进一步提高老年AML患者的缓解率及远期生存。目前研究较多的是BCL-2抑制剂VEN，多项研究显示：VEN联合去甲基化药物治疗老年初诊AML患者缓解率高，安全性良好[9]–[11]。该治疗方案已经写入美国国立综合癌症网络（NCCN）及中国AML治疗指南，成为老年不适合强化疗初诊AML患者的一线选择之一。但由于经济情况等因素，部分患者无法应用VEN为基础的治疗方案，其他可替代方案成为目前老年AML研究的方向之一。除新型靶向药物外，传统化疗药物HHT是一种细胞周期非特异性药物，可导致白血病细胞停滞在细胞周期的G_1_/G_2_期，Ara-C则作用于细胞周期的S期发挥诱导细胞凋亡的作用，HHT与Ara-C可协同诱导白血病细胞发生凋亡，而G-CSF可促使G_0_期细胞进入G_1_期，能够更有效地发挥HHT与Ara-C的协同作用[12]。此外，HHT与AZA也具有协同作用，且小剂量HHT不良反应较其他化疗药物相对较小。因此，AZA联合小剂量HHT有望进一步改善老年AML的预后。中国医学科学院血液病医院侯降雪等[13]已证实了AZA+HHT+Ara-C方案治疗AML的有效性，我科结合中国老年AML患者的实际情况，采用AZA+HAG及AZA+VEN为基础的方案诱导化疗，以期为初治老年AML患者选择更优的治疗方案提供临床依据。

我们对两种方案的疗效进行了观察，两种方案的诱导缓解率较高，AZA+VEN组患者的中位年龄及高危患者比例均高于AZA+HAG组，是影响患者预后的主要因素。AZA+VEN组1年OS率为75.8％，1年RFS率为69.0％；AZA+HAG组1年OS率为53.6％，1年RFS率为52.0％。AZA+HAG组生存较AZA+VEN组差，但差异无统计学意义，考虑可能与随访时间较短，病例偏少有关。AZA+HAG组生存相对较差主要与其复发率较高相关，AZA+HAG的高初始治疗反应率并未转化为OS的优势，可能与巩固化疗方案较弱、未持续应用AZA+HAG巩固化疗、因化疗后骨髓抑制期较长致化疗周期延长等因素相关。AZA+VEN组生存更具优势，血液学不良反应更少，说明AZA+VEN较化疗方案仍存在一定优势。本研究发现，AZA+HAG组与AZA+VEN组在总CR/CRi率、1个疗程CR/CRi率、1个疗程MRD阴性率及早期死亡率方面相似，同时不良反应均可耐受。目前AZA+VEN仍为老年AML优选治疗方案，但由于AZA+VEN治疗费用相对较高，许多患者无法负担，如患者存在经济因素或不愿意选用VEN，可考虑疗效接近的AZA+HAG方案，实际工作中，对于老年AML需根据患者年龄、身体状况、经济情况等综合考虑从而选择更适合的治疗方案。
